# Dietary Intervention Modulates the Expression of Splicing Machinery in Cardiovascular Patients at High Risk of Type 2 Diabetes Development: From the CORDIOPREV Study

**DOI:** 10.3390/nu12113528

**Published:** 2020-11-17

**Authors:** Mercedes del Río-Moreno, Raúl M. Luque, Oriol A. Rangel-Zúñiga, Emilia Alors-Pérez, Juan F. Alcalá-Diaz, Irene Roncero-Ramos, Antonio Camargo, Manuel D. Gahete, José López-Miranda, Justo P. Castaño

**Affiliations:** 1 Maimonides Institute for Biomedical Research of Cordoba (IMIBIC), 14004 Córdoba, Spain; mercedesdelriomoreno@gmail.com (M.d.R.-M.); bb2razuo@uco.es (O.A.R.-Z.); b12alpee@uco.es (E.A.-P.); jfalcala@gmail.com (J.F.A.-D.); irene_rr20@hotmail.com (I.R.-R.); antonio.camargo@imibic.org (A.C.); 2 Department of Cell Biology, University of Córdoba, 14004 Córdoba, Spain; 3 Reina Sofia University Hospital, 14004 Córdoba, Spain; 4 CIBER Fisiopatología de la Obesidad y Nutrición (CIBERobn), 14004 Córdoba, Spain; 5 Lipid and Atherosclerosis Unit, Department of Medicine, Reina Sofia University Hospital, University of Córdoba, 14004 Córdoba, Spain

**Keywords:** Low-Fat diet, Mediterranean diet, peripheral blood mononuclear cells (PBMCs), splicing machinery, Type-2 diabetes mellitus

## Abstract

Type-2 diabetes mellitus (T2DM) has become a major health problem worldwide. T2DM risk can be reduced with healthy dietary interventions, but the precise molecular underpinnings behind this association are still incompletely understood. We recently discovered that the expression profile of the splicing machinery is associated with the risk of T2DM development. Thus, the aim of this work was to evaluate the influence of 3-year dietary intervention in the expression pattern of the splicing machinery components in peripheral blood mononuclear cells (PBMCs) from patients within the CORDIOPREV study. Expression of splicing machinery components was determined in PBMCs, at baseline and after 3 years of follow-up, from all patients who developed T2DM (Incident-T2DM, *n* = 107) and 108 randomly selected non-T2DM subjects, who were randomly enrolled in two healthy dietary patterns (Mediterranean or low-fat diets). Dietary intervention modulated the expression of key splicing machinery components (i.e., up-regulation of *SPFQ*/*RMB45*/*RNU6*, etc., down-regulation of *RNU2*/*SRSF6*) after three years, independently of the type of healthy diet. Some of these changes (*SPFQ*/*RMB45*/*SRSF6*) were associated with key clinical features and were differentially induced in Incident-T2DM patients and non-T2DM subjects. This study reveals that splicing machinery can be modulated by long-term dietary intervention, and could become a valuable tool to screen the progression of T2DM.

## 1. Introduction

Type 2 diabetes mellitus (T2DM) has become a major global health problem in recent decades due to its rising incidence, prevalence, and its tight causal association with diverse comorbidities, including cardiovascular disease (CVD) [1,2]. In the context of CVD prevention, lifestyle and behavioral interventions have been shown as advantageous approaches with less associated costs and side effects compared to available medical treatments [3]. In particular, several healthy dietary patterns have been shown to be useful tools for the management of T2DM and the concomitant reduction of cardiovascular risk. This is the case of the Mediterranean diet (Med diet), Dietary Approaches to Stop Hypertension (DASH), vegetarian diet, and the low-fat (LF) high carbohydrates diet recommended by the National Cholesterol Education Program and the American Diabetes Association (ADA) [2,3,4,5]. In line with this, our group has shown that within the Coronary Diet Intervention with Olive Oil and Cardiovascular Prevention (CORDIOPREV) study, a prospective, randomized, controlled trial that includes CVD patients at high-risk of T2DM development [6], long-term consumption of a Med diet rich in olive oil and a LF diet have beneficial effects on the patients, improving insulin sensitivity and beta-cell function [7]. In this context, early identification of patients at higher risk of T2DM development is critical for prevention of new cardiovascular events [8,9]. Interestingly, in searching for novel associated molecular mechanisms and predictive markers, we recently discovered that the expression pattern of certain splicing machinery elements in the peripheral blood mononuclear cells (PBMCs) of patients is tightly associated with the risk of T2DM and could accurately predict T2DM development in those individuals from the CORDIOPREV study, outperforming the capacity of classical predictors of T2DM development, such as glycated hemoglobin (HbA1c) or predictive scores (FINDRISK) [10], two established strategies that have limitations and cannot precisely predict an individual’s risk of developing T2DM [11,12].

The splicing machinery comprises the spliceosome, a sophisticated macromolecular complex with a functional core comprising several small nuclear ribonucleoprotein (snRNP) subunits, which interact dynamically to control the splicing process. The activity of the spliceosome is precisely modulated by more than 300 auxiliary proteins, the so-called splicing factors, that recognize specific sequences in exons and introns [13,14]. An emerging body of evidence indicates that, under adverse health conditions, there is a profound dysregulation of certain spliceosomal components and splicing factors that results in altered and even aberrant splicing processes which, in turn, substantially contribute to the development of severe pathologies, including cancer, neurodegeneration, liver disease and diabetes [13,15,16,17,18,19,20,21]. Indeed, the correct function of the splicing machinery is essential to maintain cell homeostasis [17,18,22,23]. In this scenario, it has been proposed that nutrients can influence processes essential for cell homeostasis by altering gene expression and, in particular, modulating the splicing of pre-mRNAs encoding key regulatory proteins (e.g., leptin receptor, insulin receptor) [24]. Moreover, several studies have shown that the gene expression pattern of PBMCs is severely influenced by the diet [25,26,27,28] and might reflect metabolic and immune responses of adipocytes or hepatocytes [29,30], thus providing valuable information to advance in the study of diseases such as T2DM and CVDs, using less invasive sampling methods [10,27].

Based on all the above, the aim of this work was to evaluate the influence of the dietary intervention in the expression pattern of the components of the splicing machinery in PBMCs from patients included in the CORDIOPREV study. Specifically, we sought to ascertain if the consumption of two healthy diets (Med diet and LF diet) during three years modified the expression profile of the splicing machinery in PBMCs from CVD patients.

## 2. Materials and Methods

### 2.1. Study Population

The present study was conducted within the framework of the CORDIOPREV study (Clinical Trials Registry NCT0092493741), a prospective, randomized, controlled trial that includes 1002 CVD patients, who had their last coronary event over six months before joining the study [6]. Patients gave written informed consent to participate in the trial and the study protocol was approved by the Human Investigation Review Committee of the Reina Sofia University Hospital (HURS, Cordoba, Spain; (03/09/2009 session), according to institutional and Good Clinical Practice guidelines (following the Helsinki declaration). Previous reports have provided details of the study, i.e., inclusion and exclusion criteria and cardiovascular risk factors of the patients. In brief, eligible patients were 20–75 years of age, with established CVD but without clinical events in the last 6 months with no other serious diseases and a life expectancy of at least 5 years. Participants were randomly enrolled in 2 study dietary models: the Mediterranean diet (Med diet) and the low-fat diet (LF diet), a high-complex carbohydrate diet recommended by the National Cholesterol Education Program and the ADA, both providing a wide variety of foods.

At baseline, 462 participants did not present T2DM [7] and, after a median follow-up of 60 months, 107 from those patients developed T2DM (Incident-T2DM cases) (Supplementary Figure S1). Each year, T2DM was diagnosed according to the diagnosis criteria of ADA, specifically, if one or more of the following criteria were present in the study subjects: fasting plasma glucose (FPG) concentration ≥126 mg/dL, FPG ≥200 mg/dL after 2-h of oral glucose test (OGTT), glycated hemoglobin (HbA1c) ≥6.5% (≥48 mmol/mol). In the present study, we included the Incident-T2DM cases after a median follow-up of 60 months (*n* = 107) together with 108 matched, randomly selected controls from the remaining 355 subjects who did not develop T2DM (non-T2DM subjects) during the study period, as previously reported [10] (Supplementary Figure S1). The random selection of the non-T2Dm subjects was performed using stratified sampling from the 462 non-T2DM subjects of the CORDIOPREV study according to the following clinical, anthropometric and biochemical variables: diet, age, gender, fasting plasma glucose, body mass index, low-density lipoprotein (LDL)-cholesterol and high-density lipoprotein (HDL)-cholesterol. In this type of sampling, the target population was first divided into separate strata and then, samples were randomly selected within each stratum through simple sampling (1:1). These calculations were made using R Software [10]. We collected PBMC samples at baseline and at the third year of follow-up, as established in the study protocol guidelines [6].

### 2.2. Study Diets

The Med diet was composed of a minimum of 35% calories from fat (22% monounsaturated fatty acid (MUFA), 6% polyunsaturated fatty acid (PUFA) and 10% saturated fat), 15% proteins, and a maximum of 50% carbohydrates; and the LF diet by 30% total fat (12–14% MUFA, 6–8% PUFA 10% and 10% saturated fat), 15% protein, and a minimum of 55% carbohydrates. In both diets, the cholesterol content was adjusted to 300 mg/d. Participants received the same intensive dietary counseling and were monitored by nutritionists, dietitians, internists and cardiologists. Details about diets and randomization have been previously reported and summarized [31]. In the present study, 93 patients had been assigned to the LF diet group and 122 to the Med diet group (Supplementary Figure S1).

### 2.3. Metabolic Study Design

Patient metabolic status was dynamically determined by implementing OGTTs in all patients. Blood samples were taken at 30, 60, 90 and 120 min and metabolic parameters were biochemically determined, and insulin resistance and sensitivity indexes were calculated as described elsewhere [6,10].

### 2.4. Blood Sampling and Processing to Isolate Peripheral Blood Mononuclear Cells (PBMCs)

Venous blood from the participants at the inclusion of the study and after three years of follow-up (12 h overnight fast) was collected in tubes containing ethylenediaminetetraacetic (EDTA). PBMCs were isolated as reported elsewhere [6,28].

### 2.5. RNA Extraction and Quantification

Total RNA was isolated from PBMCs by using a Direct-zol RNA kit (Zymo Research, Irvine, CA, USA) following the manufacturer’s instructions. The amount of RNA recovered was determined and its quality assessed by the NanoDrop2000 spectrophotometer (Thermo Fisher, Waltham, MA, USA). One μg of RNA was reverse transcribed to cDNA using random hexamer primers with the First Strand Synthesis Kit (Thermo Fisher).

### 2.6. Analysis of Splicing Machinery Components by Microfluidic-Based Dynamic Quantitative Polymerase Chain Reaction (qPCR) Array

To determine the expression of 48 transcripts in 48 samples simultaneously, we employed a 48.48 Dynamic Array based on microfluidic technology (Fluidigm, San Francisco, CA, USA). To this end, we designed and validated specific primers for human transcripts including components of the major (*n* = 13) and minor spliceosomes (*n* = 4), associated splicing factors (*n* = 28) and three housekeeping genes, as previously detailed elsewhere [10]. Preamplification, exonuclease treatment and a quantitative polymerase chain reaction (qPCR) dynamic array were implemented using the Biomark System and the Real-Time PCR Analysis Software (Fluidigm), following the instructions of the manufacturer. A detailed description of the methodology is available in [10].

### 2.7. Statistical and Bioinformatical Analysis

Data were assessed for normality of distribution using the Kolmogorov–Smirnov test and are expressed as mean ± standard error of the mean (SEM). Statistical analysis was carried out using a paired Student’s *t*-test for the alteration of the expression pattern in each patient over the three years of the study, an unpaired *t*-test (Mann–Whitney U test) when comparing values at baseline or third year, or a Kruskal–Wallis test depending on the existence of ≥2 groups in each comparison. We studied the statistical effects of the diet ingested, independent of time, the effect of time, and the interaction of both factors. Significant correlations were studied using bivariate Spearman correlation methods; for these analyses, the fold change between the third year and baseline gene expression or biochemical parameters were calculated, in order to determine dynamic correlations. *p*-values smaller than 0.05 were considered statistically significant. Statistical analyses were carried out with GraphPad Prism 6 (La Jolla, CA, USA) and SPSS 17.0 (IBM, New York, NY, USA).

## 3. Results

### 3.1. Dietary Intervention Modulated the Expression of Several Splicing Machinery Components

**Figure 1:**
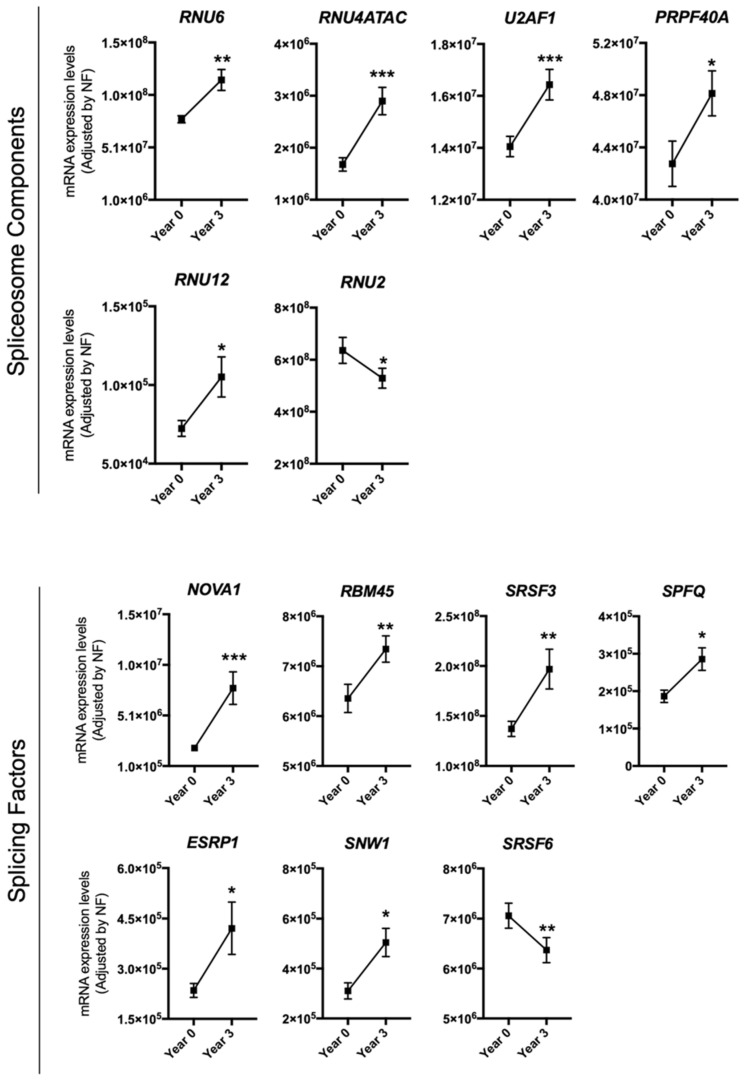
Peripheral blood mononuclear cells (PBMCs) expression pattern of specific splicing machinery components after three years of follow-up. mRNA expression levels (adjusted by a normalization factor (NF) calculated from the expression level of *GAPDH* and *ACTB*) of specific spliceosome components and splicing factors in the PBMCs from all participants included in the study. Values represent the mean ± standard error of the mean (SEM). Asterisks indicate values that significantly differ from non-type 2 diabetes mellitus (T2DM) subjects (*t*-test: *, *p* < 0.05; **, *p* < 0.01; ***, *p* < 0.001).

The effect of dietary intervention on the expression pattern of splicing machinery components was evaluated in PBMCs from 215 CVD patients at high risk of T2DM development included in the CORDIOPREV study. Microfluidic-based qPCR analysis revealed that the expression pattern of several splicing machinery components was altered in PBMCs from these patients after three years of dietary intervention. Specifically, results unveiled an increase in the expression of the spliceosome components *RNU6, RNU4ATAC, U2AF1, PRPF40A,* and *RNU12* and in the splicing factors *NOVA1, SRSF3, RBM45, SPFQ, ESRP1*, and *SNW1*, as well as a decrease of the spliceosome component *RNU2* and the splicing factor *SRSF6* (Figure 1). Further analysis indicated that the increase in the expression of *SPFQ* during the 3 years of follow-up observed herein was inversely correlated with the evolution of the homeostatic model assessment-insulin resistance (HOMA-IR) and hepatic insulin resistance index (HIRI) indexes (Table 1), which decreased during the follow-up in the study population (Supplementary Table S1). Moreover, *RBM45* levels at year 3 of follow-up were inversely correlated with HOMA-IR and HIRI at this time point (Table 2).

### 3.2. The Modulation of the Expression of Most Splicing Machinery Components Was Not Diet-Dependent

**Figure 2:**
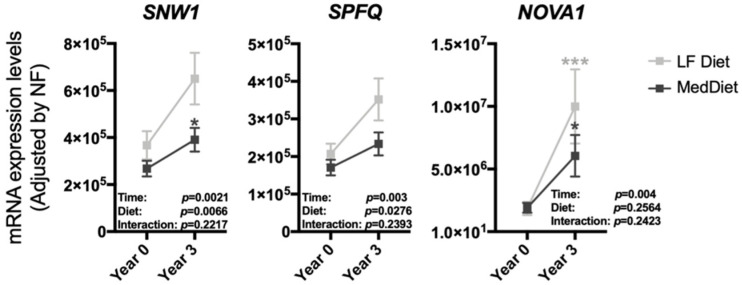
PBMCs expression pattern of the specific splicing machinery components after three years of follow-up under two healthy dietary patterns (low-fat (LF) diet and Mediterranean (Med) diet). mRNA expression levels (adjusted by a normalization factor (NF) calculated from the expression level of *GAPDH* and *ACTB*) of specific spliceosome components and splicing factors in the PBMCs from all the participants included in the study. Values represent the mean ± SEM. Asterisks indicate values that significantly differ from non-T2DM subjects (*t*-test: *, *p* < 0.05; ***, *p* < 0.001).

At baseline, all CORDIOPREV participants were randomly enrolled in one of the two dietary model groups, LF diet and Med diet, showing comparable levels of all parameters determined (Supplementary Tables S2 and S3). Interestingly, while most of the splicing machinery components altered during the dietary intervention showed similar trends and changes when the population was separated by diets (Supplementary Figure S2), a distinct, differential response (statistically significant effects for diet and time) was observed in the case of the splicing factors *SNW1*, *SPFQ* and *NOVA1*. In particular, the observed increase in the expression of these factors was more pronounced in PBMCs from patients under a LF diet than in those from Med diet patients (Figure 2).

### 3.3. The Expression Pattern of Specific Splicing Machinery Components Was Differentially Modulated by Dietary Intervention in Incident-T2DM Cases and Non-T2DM Controls

**Figure 3:**
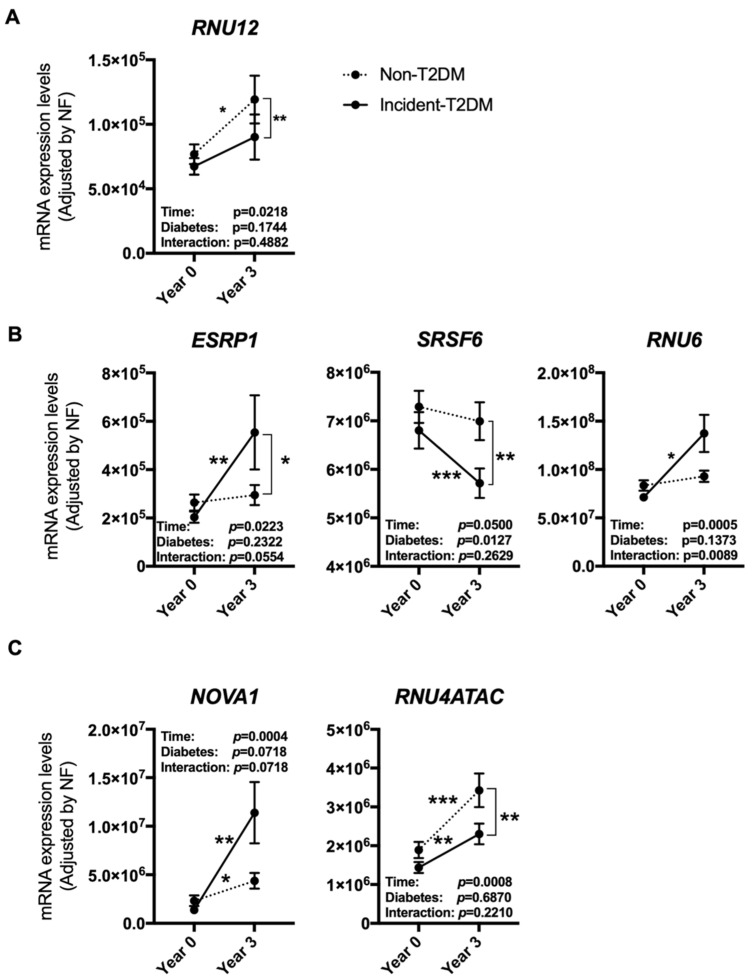
PBMCs expression pattern of specific splicing machinery components after three years of follow-up in non-T2DM and incident-T2DM subjects. Splicing machinery components altered in non-T2DM subjects (**A**), incident-T2DM subjects (**B**) or both (**C**). mRNA expression levels [adjusted by a normalization factor (NF) calculated from the expression level of GAPDH and ACTB] of specific spliceosome components and splicing factors in the PBMCs from all the participants included in the study. Values represent the mean ± SEM. Asterisks indicate values that significantly differ from non-T2DM subjects (*t*-test: *, *p* < 0.05; **, *p* < 0.01; ***, *p* < 0.001).

After dietary intervention, 107 participants had developed T2DM (Incident-T2DM) [32], while 108 participants who did not develop T2DM were considered as non-T2DM control individuals [10]. The expression of some of the previously mentioned spliceosome components and splicing factors was differentially altered in the PBMCs of patients who developed T2DM after a median follow-up of 60 months compared to non-T2DM subjects. Specifically, *RNU12* was clearly increased in non-T2DM controls as compared to incident-T2DM patients (Figure 3A). On the other hand, *ESRP1* and *RNU6* were markedly increased, and *SRSF6* reduced, in Incident-T2DM patients, while no such changes were observed in non-T2DM subjects (Figure 3B). Of note, *SRSF6* decrease was directly correlated with the decrease in HbA1c levels observed in Incident-T2DM patients during the follow-up (ρ: 0.227; *p*: 0.047). In addition, we observed that *NOVA1* and *RNU4ATAC* were significantly altered both in Incident-T2DM cases and non-T2DM controls; however, the increase of *NOVA1* was clearly more pronounced in Incident-T2DM cases, wherein it inversely correlated with HOMA-IR (ρ: −0.245; *p*: 0.028), while the increase of *RNU4ATAC* was more pronounced in non-T2DM controls (Figure 3C).

**Figure 4:**
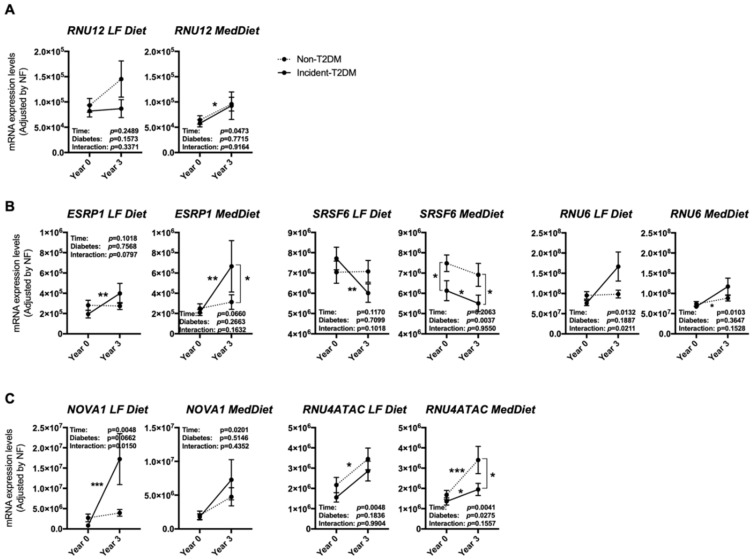
PBMCs expression pattern of specific splicing machinery components after three years of follow-up in non-T2DM and incident-T2DM subjects under two healthy dietary patterns (LF diet and Med diet). Splicing machinery components altered in non-T2DM subjects (**A**), incident-T2DM subjects (**B**) or both (**C**). mRNA expression levels (adjusted by a normalization factor (NF) calculated from the expression level of *GAPDH* and *ACTB*) of specific spliceosome components and splicing factors in the PBMCs from all the participants included in the study. Values represent the mean ± SEM. Asterisks indicate values that significantly differ from non-T2DM subjects (*t*-test: *, *p* < 0.05; **, *p* < 0.01; ***, *p* < 0.001).

Of the non-T2DM subjects, at the beginning of the study, 51 had been randomly assigned to the LF diet and 57 to the Med diet, while from Incident-T2DM patients, 42 had been initially assigned to the LF diet and 65 to the Med diet (Supplementary Figure S1 and Tables S4 and S5). When analyzing the expression pattern according to the diabetic status and the dietary group, several differences emerged between groups. In particular, *RNU12,* which was more increased in non-T2DM subjects after the three years of follow-up (Figure 3A), seemed to be especially stimulated under the LF diet, although it did not reach statistical significance (Figure 4A). On the other hand, the differences observed in the splicing factor *ESRP1* and *RNU6* between non-T2DM controls and Incident-T2DM patients were stronger under the Med diet and LF diet, respectively (Figure 4B); while changes in *SRSF6* were similar in study subjects under both dietary interventions. Finally, the increase in *NOVA1* was strikingly more pronounced under the LF diet than under the Med diet in Incident-T2DM patients, whereas the increase in *RNU4ATAC* was more obvious in the Med diet group in non-T2DM controls (Figure 4C).

## 4. Discussion

This study represents, to the best of our knowledge, the first comprehensive analysis of the regulatory role of healthy dietary interventions on the expression of the components of the splicing machinery, including spliceosome elements and splicing factors. This study was implemented using PBMCs from CVD patients at high risk of T2DM development included in the CORDIOPREV trial, in that we have previously demonstrated that the expression pattern of certain splicing machinery components is associated with the risk of T2DM development and could accurately predict this development in individuals with coronary heart disease [10].

Our present study provides primary evidence that a dietary intervention can distinctly alter the expression pattern of the splicing machinery, both spliceosome components and splicing factors, in CVD patients at high risk of T2DM. In particular, these results demonstrate that the consumption of two healthy diets (Med diet and LF diet) during three years can modulate the expression pattern of key spliceosome components and splicing factors in PBMCs from the patients enrolled in the CORDIOPREV study, including the overexpression of some molecular components, like *SPFQ*, *RBM45*, *RNU6*, etc. and the downregulation of others, including *RNU2* and *SRSF6*. Interestingly, some of the changes observed in the expression levels of certain splicing machinery components were closely associated with relevant biochemical parameters and clinical features, as is the case of the increase in the expression levels of the splicing factor *SPFQ*, which was inversely correlated with the decrease in HOMA-IR and HIRI indexes observed in the population.

The finding of a diet-related long-term modulation of the expression of the splicing machinery components could represent a novel valuable piece of information for two reasons. First, because it unveils that the splicing process may represent an adaptive mechanism in response to different nutritional conditions, and that this mechanism could be in place not only in circulating PBMCs but may also operate in cell types from other tissues and organs tightly coupled to nutrient-dependent metabolic homeostasis (e.g., liver, pancreas, adipose tissue), an avenue that is indeed worth exploring. Actually, we and others have already discovered the delicate and important role that the regulation of the splicing machinery can play in those organs [10,18,33,34,35]. Secondly, inasmuch as PBMCs can be an accessible and suitable sentinel to detect relevant changes related to nutrient- and diet-dependent metabolic homeostasis, our current results support the idea that changes in the expression of key splicing machinery components could provide a fine screening marker for the development or progression of T2DM and their diet-related dynamics. Indeed, within the CORDIOPREV study, the long-term intake of a Med diet, rich in olive oil, or a LF diet similarly improved insulin sensitivity and beta-cell function [7] and, therefore, the increase in the expression of specific splicing factors found herein under both diets, and their inverse correlation with insulin resistance indexes, strongly suggest that the molecular changes might be related to the beneficial consequence of consumption of a healthy diet. Furthermore, the dynamic, concomitant changes in expression observed suggest that splicing factors and, hence, the alternative splicing process, could represent novel elements within the complex mechanisms linking healthy dietary intervention and the improvement of patients’ metabolic status and the consequent protection from cardiovascular complications. Given the very scarce information available on the functional roles and implications of many of the molecules identified in the present study to be altered in PBMCs (e.g., *SPFQ*, *RBM45*, *RNU6*, etc.), the present findings open novel, unexplored avenues in this field of research.

One of the findings from this study that we consider most noteworthy is that the diet-induced alterations in the splicing machinery of PBMCs was independent of the type of healthy diet in which CORDIOPREV participants were enrolled (Med diet or LF diet), except for three splicing factors (*SNW1*, *SPFQ* and *NOVA1*) that showed a more pronounced modulation in patients under the LF diet. To date, and to the best of our knowledge, no data were reported regarding the influence of diet intervention in the modulation of the expression of *SNW1*, *SPFQ* and *NOVA1*. However, some of these factors have been described to contribute to the alternative splicing of key genes whose splicing processing changes in response to a fatty diet [34]. In particular, *NOVA1* expression has been shown to be modulated under high-fat diet-induced obesity and to be responsible for the regulation of the splicing process of key genes under these conditions [34]. Of note, it has been proposed that *NOVA1* is a master regulator of alternative splicing in pancreatic beta cells, where it controls the expression of key genes involved in insulin transcription and secretion [36]. Conversely, to date, the regulation and possible role of this splicing factors in PBMCs have not been explored in detail.

Previously, several studies have shown that PBMCs’ gene expression pattern is influenced by the diet [25,26,27,28] and that this might reflect changes related to both metabolic and immune responses [29,30]. In addition, it has been demonstrated that the splicing process of key regulatory proteins for metabolic homeostasis, like the receptors for insulin or leptin, can be markedly influenced by nutrient metabolism, directly or indirectly [24,37]. Thus, it seems reasonable to think that those splicing-related changes would rely on upstream changes in the function of the machinery responsible for generating the splice variants. However, little or nothing is known in this regard in PBMCs, for there are no reports on how diet can influence the expression of the components of the spliceosome and the splicing factors, which altogether are responsible of the modulation of the splicing process. Nevertheless, in this context, some studies have highlighted that nutritional status can induce changes in the activity of serine-arginine (SR) proteins [38], an important family of splicing factors, further supporting the contention that different nutrients may be able to modulate the expression of metabolic genes at the level of its splicing processing. Specifically, it has been shown that insulin signaling can up-regulate the expression of the splicing factor SRSF1 in pancreatic beta cells, inducing the splicing of the insulin receptor to generate the INSR-B isoform [39]. The same study also found a regulation of the protein levels of the splicing factor MBNL1 by high glucose levels. In addition, other splicing factors belonging to the SR proteins family, SRSF2, are decreased under a vitamin E-deficient diet in the liver [40]. Thus, although still limited, the evidence is growing, and by inclusion of the results from this study, pointing to a link between diet and nutrient and regulation of the splicing process, including its underlying operating machinery.

Another intriguing implication of our present results relates to the predictive capacity of studying changes in the splicing machinery in at-risk patients. To be more specific, nutrient-induced changes in specific splicing machinery components may provide hints on the predictive potential and possible functional correlation of key molecules, which had not been explored hitherto in this regard. Thus, within this study, regardless of the type of dietary intervention, the expression of some of the splicing factors studied was differentially altered in patients that develop T2DM after the 5 years of the study compared to non-T2DM subjects. For example, *RNU12*, a component of the minor spliceosome, showed a significant increase after the 3 years of dietary intervention in non-T2DM controls. Interestingly, we have previously described how the expression of this small nuclear RNA (snRNA), which is essential to form U12 snRNP and carry out the appropriate splicing of type 12 introns [41], was lower, at baseline (inclusion of the study), in Incident-T2DM compared to non-T2DM subjects and that this was associated with the risk of T2DM development [10]. Therefore, since lower expression levels of *RNU12* were associated with a higher risk of T2DM, it is reasonable to infer that the dietary-induced increase in the expression of this component in non-T2DM might be related to the protective effects of the healthy dietary consumption. In support to this notion, in that same study, a 4 h-incubation with baseline postprandial serum from Incident-T2DM patients induced a significant reduction of *RNU12* expression compared to non-T2DM treated PBMCs from healthy patients [10]. Therefore, from a more general standpoint, it can be proposed that modulation of the expression of specific spliceosomal components may represent a link between dietary intervention and beneficial effects on the patient metabolic status. Remarkably, the difference in *RNU12* expression, at year 3, between incident patients and non-T2DM subjects, was more pronounced under the LF diet. Thus, although the possible mechanisms linking nutrient-induced changes in the splicing machinery, their functional consequences and the regulatory implications are still to be fully elucidated, our present study provides supportive evidence to further explore both the mechanistic/functional and the predictive components of this plausible link, for it may provide original, valuable biological knowledge as well as practical information for the patients.

This study has strengths and limitations. Among the latter, it should be mentioned that (1) the analyses of the expression patterns of key splicing machinery elements was carried out in the heterogeneous PBMC population, but not in specific cell types; (2) the sample sizes of T2DM patients and non-T2DM controls were relatively small; and (3) the non-T2DM subjects analyzed were CVD patients within the CORDIOPREV study and, therefore, were not non-T2DM healthy controls. On the other hand, one of the strengths of our work derives from its nested case-control study nature, which enabled both the T2DM patients and non-T2DM subjects to be sampled from the longitudinal CORDIOPREV study. Also, expression patterns of a wide array of spliceosome components and splicing factors were studied.

In conclusion, this study reveals that expression of the splicing machinery components in PBMCs from CVD patients at risk of T2DM can be notably and selectively influenced by long-term dietary intervention; also, that the two dietary interventions tested herein, Med diet and LF diet, induced remarkably similar changes on the expression of spliceosome components; and, finally, that there are distinct, diet type-induced changes in PBMCs from both non-T2DM and incident-T2DM patients, that may have an as yet unknown functional significance. Therefore, we propose that the machinery that controls and performs the alternative splicing process, which is consequently responsible for changes in the pattern of functionally and pathologically relevant splice variants involved in the regulation of metabolic homeostasis, is a plausible target to be operated by dietary intervention. As such, our results pave the way to explore in experimental models the possible mechanistic role and relevance of the splicing machinery and its components in diet-related metabolic regulation, and to investigate the value of screening changes in specific splicing machinery components to monitor and predict early the relevant diet-related changes in CVD patients at risk of T2DM.

## Figures and Tables

**Table 1:** Correlations between the fold change in the expression levels of splicing machinery components (3rd year/baseline levels) and the fold change in relevant T2DM related parameters.

			Fold Change during Follow-Up	
			Study Population (*n* = 215) Incident-T2DM + Non-T2DM Controls	Incident-T2DM (*n* = 107)
			*SPFQ*	*SRSF6*
Fold change during follow-up	HOMA-IR	ρ (rho)	−0.157	
		*p*	0.03 *	
	HIRI	ρ (rho)	−0.176	
		*p*	0.018 *	
	HbA1c (%)	ρ (rho)		0.227
		*p*		0.047 *

HbA1c: glycated hemoglobin; HOMA-IR: Homeostasis model assessment- insulin resistance; HIRI: Hepatic insulin resistance index; Non-significant correlations are not depicted. * indicates *p* < 0.05.

**Table 2:** Correlations between the expression levels of RBM45 and relevant T2DM related parameters at year 3 in the total study population (Incident-T2DM + non-T2DM controls *n* = 215).

			Levels at Year 3 (*n* = 215) Incident-T2DM + Non-T2DM Controls
			*RBM45*
Levels at year 3	HOMA-IR	ρ (rho)	−0.166
		*p*	0.018 *
	HIRI	ρ (rho)	−0.166
		*p*	0.018 *

HOMA-IR: Homeostasis model assessment- insulin resistance; HIRI: Hepatic insulin resistance index. * indicates *p* < 0.05. TD2M: Type 2 Diabetes Mellitus.

## Supplementary Materials

The following are available online at https://www.mdpi.com/2072-6643/12/11/3528/s1, Figure S1: Graphical scheme of the study timeline and participant selection, Figure S2: PBMCs expression pattern of specific splicing machinery components after three years of follow-up under two healthy dietary pattern (LF Diet and MedDiet), Table S1: Demographic and metabolic characteristics of the study cohort (n = 215 patients) at baseline and after three years of follow-up, Table S2: Demographic and metabolic characteristics of the study cohort (n = 215 patients) at baseline, separated by dietary intervention (Low fat diet vs. Med diet), Table S3: Demographic and metabolic characteristics of the study cohort (n = 215 patients) at baseline and after three years of follow-up, separated by dietary intervention (Low fat diet vs. Med diet), Table S4: Demographic and metabolic characteristics after three years of follow-up of Non-T2DM cases under LF Diet and Mediterranean Diet, Table S5: Demographic and metabolic characteristics after three years of follow-up of Incident-T2DM cases under LF Diet and Mediterranean Diet.
