# Supplementary material for: Dietary Intervention Modulates the Expression of Splicing Machinery in Cardiovascular Patients at High Risk of Type 2 Diabetes Development: From the CORDIOPREV Study
Source: Nutrients. 2020 Nov 17;12(11):3528. doi: 10.3390/nu12113528 (PMC7696699; doi:10.3390/nu12113528)
Supplement: Supplementary file 1 [file nutrients-12-03528-s001.pdf]

Supplementary material

Supplementary Figure S1

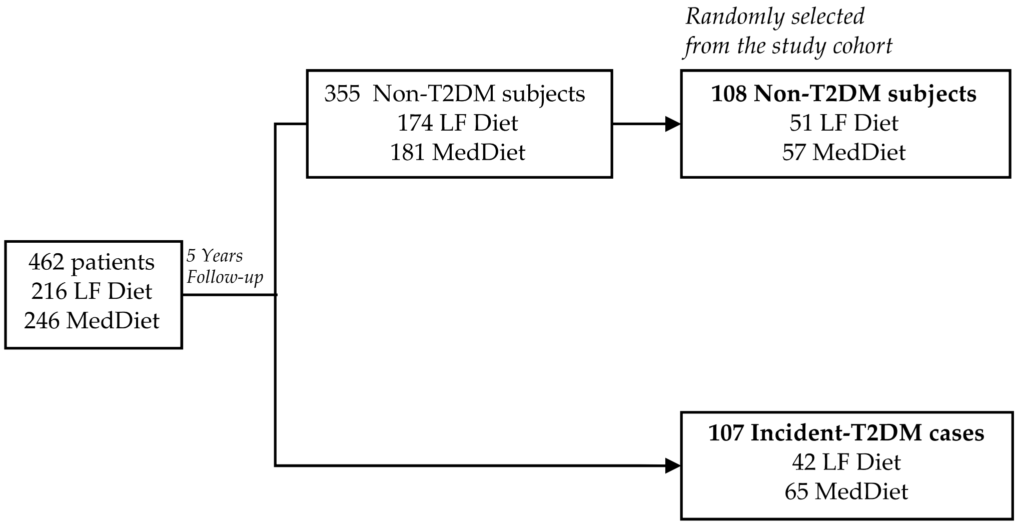

Supplementary Figure S1. Graphical scheme of the study timeline and participant selection. Specifically, from the initial 462 non-T2DM subjects included in the CORDIOPREV study, 107 patients developed T2DM (Incident-T2DM cases) after a mean follow-up of 5-years according to all the American Diabetes Association (ADA) diagnosis criteria evaluated on the basis of glucose tolerance tests (OGTT) performed each year. In the present study, all these 107 incident-T2DM cases and 108 matched controls (non-T2DM subjects, randomly selected from the remaining 355 subjects that did not develop T2DM during the study period) were included. The number of patients enrolled in each dietary pattern is included. LF Diet: Low-fat diet; MedDiet: Mediterranean diet.

Supplementary Figure S2

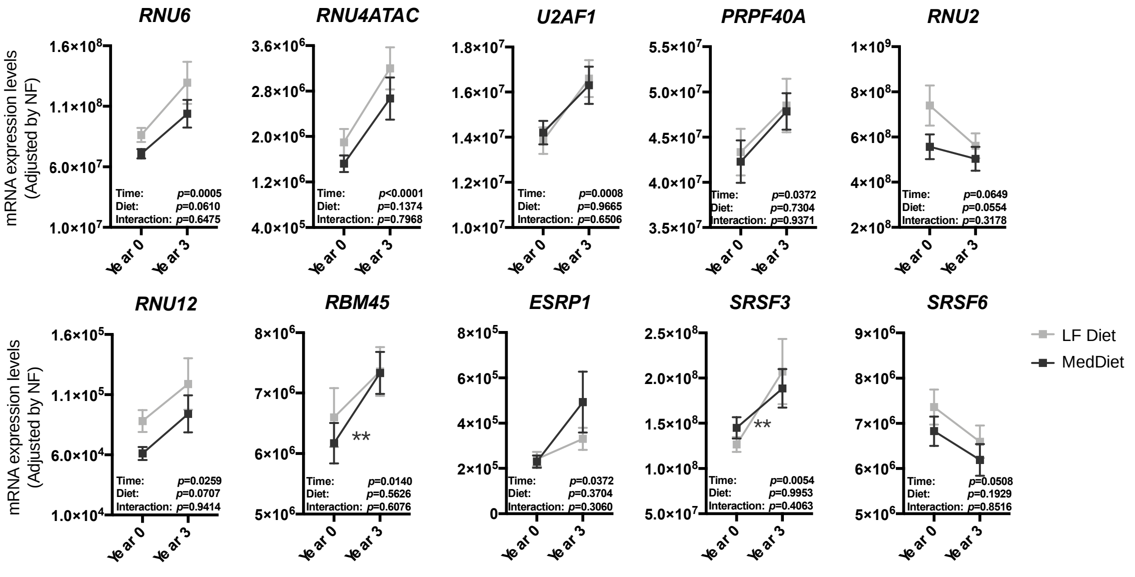

Supplementary Figure S2. PBMCs expression pattern of specific splicing machinery components after three years of follow-up under two healthy dietary pattern (LF Diet and MedDiet). mRNA expression levels [adjusted by a normalization factor (NF) calculated from the expression level of *GAPDH* and *ACTB*] of specific spliceosome components and splicing factors in the PBMCs from all the patients included in the study. Values represent the mean ± SEM. Asterisks indicate values that significantly differ from non-T2DM subjects (t-test: **, p<0.01). LF Diet: Low-fat Diet; MedDiet: Mediterranean Diet.

Supplementary Table S1

	Basal (mean ± SEM)	YEAR 3 (mean ± SEM)	*p*-value
Weight (kg)	83.10 ± 0.91	82.61 ± 0.98	0.3327
BMI	30.61 ± 0.29	30.40 ± 0.31	0.1612
Waist circumference (cm)	103.54 ± 0.71	103.23 ± 0.74	0.5523
HbA1c (%)	5.94 ±0.02	5.60 ± 0.03	0.0000
Glucose (mg/dL)	95.25 ± 0.71	99.06 ± 0.76	0.0000
Insulin (mU/L)	9.32 ± 0.44	9.43 ± 0.56	0.8514
HIRI	1184.85 ± 68.58	955.78 ± 61.76	0.0013
HOMA-IR	2.94 ± 0.17	2.37 ± 0.15	0.0011

Supplementary Table S1. Demographic and metabolic characteristics of the study cohort (n=215 patients) at baseline and after three years of follow-up. Values expressed as mean ± SEM. BMI: Body mass index; HbA1c: Glycated hemoglobin; HIRI: Hepatic insulin resistance index; HOMA-IR: Homeostasis model assessment-insulin resistance.

Supplementary Table S2

	Low Fat diet (mean ± SEM)	Med diet (mean ± SEM)	p-value
Weight (Kg)	84.3 ± 1.6	83.4 ± 1.2	0.6642
BMI	30.9 ± 0.5	30.7 ± 0.4	0.7164
Waist circumference (cm)	104.5 ± 1.2	103.4 ± 0.9	0.4841
HbA1c (%)	6.0 ± 0.0	5.9 ± 0.0	0.2102
Glucose (mg/dL)	94.5 ± 1.1	95.8 ± 0.9	0.3650
Insulin (mU/L)	9.0 ± 0.6	9.5 ± 0.6	0.6122
HIRI	1145.9 ± 76.1	1235.2 ± 101.5	0.5061
HOMA-IR	2.9 ± 0.2	3.0 ± 0.3	0.7546

Supplementary Table S2. Demographic and metabolic characteristics of the study cohort (n=215 patients) at baseline, separated by dietary intervention (Low fat diet vs. Med diet). Values expressed as mean ± SEM. BMI: Body mass index; HbA1c: Glycated hemoglobin; HIRI: Hepatic insulin resistance index; HOMA-IR: Homeostasis model assessment- insulin resistance.

Supplementary Table S3

	Baseline		3 years follow-up				
	Low Fat diet (mean SEM)	Med diet (mean SEM)	Low Fat diet (mean SEM)	Med diet (mean SEM)	p Time	p Diet	p Time vs Diet
Weight (kg)	84.11 1.50	83.93 1.29	82.55 1.53	83.56 1.32	0.01	0.83	0.111
BMI	30.93 0.46	30.75 0.39	30.36 0.48	30.63 0.41	0.013	0.94	0.1
Waist circumference (cm)	104.28 1.12	103.21 0.97	103.99 1.15	103.04 0.99	0.58	0.48	0.88
HbA1c (%)	5.98 0.04	5.91 0.03	5.58 0.04	5.62 0.04	<0.01	0.77	0.02
Glucose (mg/dL)	94.79 1.08	95.62 0.95	97.46 1.14	100.32 1.01	<0.01	0.16	0.15
Insulin (mU/L)	8.91 0.67	9.65 0.59	8.41 0.83	10.24 0.74	0.94	0.12	0.34
HIRI	1136 103	1223 92	854 93	1036 82	<0.01	0.22	0.50
HOMA-IR	2.87 0.26	2.98 0.23	2.04 0.23	2.63 0.20	<0.01	0.21	0.16

Supplementary Table S3. Demographic and metabolic characteristics of the study cohort (n=215 patients) at baseline and after three years of follow-up, separated by dietary intervention (Low fat diet vs. Med diet). Values expressed as mean ± SEM. BMI: Body mass index; HbA1c: Glycated hemoglobin; HIRI: Hepatic insulin resistance index; HOMA-IR: Homeostasis model assessment- insulin resistance.

Supplementary Table S4

	Non-T2DM						
	Baseline		3 years follow-up				
	Low Fat diet (mean SEM)	Med diet (mean SEM)	Low Fat diet (mean SEM)	Med diet (mean SEM)	p Time	p Diet	p Time vs Diet
Weight (kg)	82.89 1.77	81.04 1.66	81.21 2.05	81.72 1.92	0.525	0.79	0.132
BMI	30.44 0.54	29.99 0.51	29.80 0.62	29.93 0.58	0.043	0.83	0.094
Waist circumference	103.46 1.36	100.24 1.28	102.49 1.50	100.52 1.41	0.53	0.172	0.25
HbA1c (%)	5.91 0.05	5.84 0.04	5.49 0.05	5.58 0.05	<0.01	0.82	0.02
Glucose (mg/dL)	94.44 1.36	94.70 1.29	95.24 1.34	97.77 1.27	0.04	0.39	0.22
Insulin (mU/L)	7.81 0.76	7.96 0.71	7.81 0.87	7.33 0.82	0.68	0.84	0.69
HIRI	1034 75.70	1013 71.53	795 89.88	718 84.93	<0.01	0.59	0.69
HOMA-IR	2.61 0.19	2.47 0.18	1.81 0.18	1.77 0.17	<0.01	0.68	0.72

Supplementary Table S4. Demographic and metabolic characteristics after three years of follow-up of Non-T2DM cases under LF Diet and Mediterranean Diet. Values expressed as mean ± SEM. BMI: Body mass index; HbA1c: Glycated hemoglobin; HIRI: Hepatic insulin resistance index; HOMA-IR: Homeostasis model assessment- insulin resistance.

Supplementary Table S5

	INCIDENT-T2DM						
	Baseline		3 years follow-up				
	Low Fat diet (mean SEM)	Med diet (mean SEM)	Low Fat diet (mean SEM)	Med diet (mean SEM)	p Time	p Diet	p Time vs Diet
Weight (kg)	85.6 2.4	85.5 1.9	84.2 2.3	85.2 1.8	0.142	0.87	0.388
BMI	31.5 0.7	31.4 0.6	31.0 0.7	31.3 0.6	0.132	0.95	0.46
Waist circumference	105.3 1.8	105.8 1.4	105.8 1.7	105.2 1.4	0.98	0.995	0.40
HbA1c (%)	6.1 0.1	6.0 0.0	5.7 0.1	5.7 0.1	<0.01	0.36	0.34
Glucose (mg/dL)	95.2 1.7	96.5 1.4	100.2 1.9	102.7 1.5	<0.01	0.36	0.56
Insulin (mU/L)	10.3 1.1	11.2 0.9	9.1 1.4	13.0 1.2	0.71	0.09	0.10
HIRI	1261 201.1	1423 167.6	926 162.4	1339 135.4	0.1	0.16	0.33
HOMA-IR	3.2 0.5	3.5 0.4	2.3 0.4	3.4 0.3	0.167	0.17	0.19

Supplementary table S5. Demographic and metabolic characteristics after three years of follow-up of Incident-T2DM cases under LF Diet and Mediterranean Diet. Values expressed as mean ± SEM. BMI: Body mass index; HbA1c: Glycated hemoglobin; HIRI: Hepatic insulin resistance index; HOMA-IR: Homeostasis model assessment- insulin resistance.
